# Lactoferrin: A Natural Glycoprotein Involved in Iron and Inflammatory Homeostasis

**DOI:** 10.3390/ijms18091985

**Published:** 2017-09-15

**Authors:** Luigi Rosa, Antimo Cutone, Maria Stefania Lepanto, Rosalba Paesano, Piera Valenti

**Affiliations:** 1 Department of Public Health and Infectious Diseases, University of Rome La Sapienza, 00185 Rome, Italy; luigi.rosa@uniroma1.it (L.R.); antimo.cutone@uniroma1.it (A.C.); mariastefania.lepanto@uniroma1.it (M.S.L.); 2 Department of Gynecological-Obstetric and Urological Sciences, University of Rome La Sapienza, 00185 Rome, Italy; rosalba.paesano@uniroma1.it

**Keywords:** lactoferrin, iron, inflammation, anemia, oral care, cytokines, athletes, homeostasis

## Abstract

Human lactoferrin (hLf), an iron-binding multifunctional cationic glycoprotein secreted by exocrine glands and by neutrophils, is a key element of host defenses. HLf and bovine Lf (bLf), possessing high sequence homology and identical functions, inhibit bacterial growth and biofilm dependently from iron binding ability while, independently, bacterial adhesion to and the entry into cells. In infected/inflamed host cells, bLf exerts an anti-inflammatory activity against interleukin-6 (IL-6), thus up-regulating ferroportin (Fpn) and transferrin receptor 1 (TfR1) and down-regulating ferritin (Ftn), pivotal actors of iron and inflammatory homeostasis (IIH). Consequently, bLf inhibits intracellular iron overload, an unsafe condition enhancing in vivo susceptibility to infections, as well as anemia of inflammation (AI), re-establishing IIH. In pregnant women, affected by AI, bLf oral administration decreases IL-6 and increases hematological parameters. This surprising effect is unrelated to iron supplementation by bLf (80 μg instead of 1–2 mg/day), but to its role on IIH. AI is unrelated to the lack of iron, but to iron delocalization: cellular/tissue overload and blood deficiency. BLf cures AI by restoring iron from cells to blood through Fpn up-expression. Indeed, anti-inflammatory activity of oral and intravaginal bLf prevents preterm delivery. Promising bLf treatments can prevent/cure transitory inflammation/anemia/oral pathologies in athletes.

## 1. Iron and Its Homeostasis

Iron, an essential element for cell growth and proliferation, is a component of fundamental processes such as DNA replication and energy production. However, iron can also be toxic when present in excess for its capacity to donate electrons to oxygen, thus causing the generation of reactive oxygen species (ROS), such as superoxide anions and hydroxyl radicals [1]. ROS are known to cause tissue injury and organ failure by damaging a number of cellular components, including DNA, proteins and membrane lipids. This dichotomy of iron, able to gain and loss electrons, has led to the evolution of tight controls on iron uptake to minimize iron deficiency, as well as iron excess. Sophisticated strategies have been developed both to avoid iron in free available toxic form and to maintain the correct iron balance/ratio between tissues/secretions and blood, defined as iron homeostasis.

In humans, total body iron, about 3 g for women and 4 g for men, is distributed in two main forms: hemic-iron, mostly found in the hemoglobin, myoglobin and cytochromes (2–2.7 g), and non-hemic-iron, a cofactor of several enzymes. Dietary iron is absorbed in the proximal small intestine (duodenum). In developed countries, about 15 mg of iron per day are provided by a balanced diet, but only ~10% (1–2 mg) is absorbed, due to its extremely poor bio-availability. Interestingly, 20 mg of iron per day, used for the de novo synthesis of heme, derive from senescent erythrocyte lyses by macrophages. The iron recovered from hemoglobin of senescent erythrocytes is the largest iron source in the reticuloendothelial system. Finally, every day, a few milligrams of iron are regained from storage in hepatocytes and macrophages. In human cells, the required iron is guaranteed by transferrin (Tf)-bound iron, which is imported into cells through receptor-mediated endocytosis. In the endosome, Tf-bound iron is released as ferrous ion, which is translocated via divalent metal transporter 1 (DMT1) into cytoplasm where it is sequestered by ferritin (Ftn). Ftn, the major iron storage protein, composed by 24 subunits, possesses ferroxidase activity and a large cavity where up to 4500 ferric ions, as oxy-hydroxide micelles, are sequestered. The release of iron from this protein to cytoplasm occurs after reduction of ferric to ferrous ions. Then, ferrous ions are exported into plasma by ferroportin (Fpn), the only known mammalian iron exporter found on the cytoplasmic membrane of enterocytes, hepatocytes, macrophages and placental cells [2]. Of note, Fpn acts in partnership with two ferroxidases: hephaestin (Heph), found in epithelial cells, and ceruloplasmin (Cp), in macrophages [3]. Both ferroxidases convert ferrous into ferric ions in order to allow their binding to Tf in the blood.

Fpn is an important actor of iron homeostasis, regulated by multiple factors. In particular, Fpn is down-regulated by the pro-inflammatory cytokine interleukin-6 (IL-6) [4,5] and by hepcidin, another pivotal actor, which regulates iron homeostasis through the binding, internalization and degradation of Fpn [6]. The bioactive hepcidin, a cationic peptide hormone of 25 amino acids mainly synthesized by hepatocytes, derives from the proteolytic cleavage of an 84-amino acid precursor, and it is secreted in urine [7,8] and plasma [9]. As Fpn, hepcidin is controlled by several factors. In particular, it is transcriptionally feedback-regulated by iron stores [10]. This mechanism involves multiple pathways through which hepatocytes directly sense systemic iron levels [10,11]. Hepcidin synthesis is also up-regulated by pro-inflammatory cytokines, such as IL-6, IL-1α and IL-1β [12,13,14,15]. The Fpn degradation caused by the binding with hepcidin or its down-regulation by IL-6 provokes a significant decrease of iron export from cells into plasma. Consequently, at the cellular level, iron overload in the host cells including enterocytes and macrophages is established, while at the systemic level, iron deficiency (ID), ID anemia (IDA) and anemia of inflammation (AI) have been found [16,17]. In ID without anemia, total serum iron (TSI) concentration and serum Ftn (sFtn) are very low, while hemoglobin (Hb) levels remain normal. ID may be classified according to sFtn and TSI concentrations (<24 ng/mL and <30 mg/dL, respectively) as mild (sFtn = 12–24 ng/mL) or severe ID (sFtn < 12 ng/mL). In IDA, the iron stores are low or absent, resulting in severe low hemoglobin (Hb) and red blood cell (RBC) levels. IDA is usually classified in line with the number of RBCs (<4 × 10^6^ cells/mL) and Hb concentration (<11 g/dL,) as mild (Hb 7–10.9 g/dL) or severe IDA (Hb < 7 g/dL). In AI, in addition to the low hematological parameters, normal-to-elevated sFtn and high levels of IL-6 and other pro-inflammatory cytokines are observed.

ID, IDA and AI are the most widespread iron disorders with over 30% of the world’s population affected by anemia, with the cause of anemia primarily due to iron deficiency [18]. In particular, in developing countries, the high amounts of polyphenols (tannins) and phytates, present in cereal- and tuber-based diets, increase ID and IDA incidence due to their influence on iron absorption. The most common intervention programs for ID and IDA are based on the classical preconception that oral iron administration or the consumption of iron-rich foods increases hematological parameters, thus reducing the prevalence of anemia. However, although most of these programs designed by governments and international agencies are easy to perform at low cost, they are ineffective in preventing and curing ID and IDA. Several studies highlighted how ferrous iron supplementation often fails to restore iron homeostasis disorders in patients suffering from ID and IDA, causing frequent adverse effects, such as gastrointestinal discomfort, nausea, vomiting, diarrhea and constipation [19,20,21,22,23]. Moreover, in vivo studies in rats fed with an iron-enriched diet showed a higher production of ROS [24] and a more severe progression of the colitis inflammatory status [25].

AI, the most severe iron homeostasis disorder, is difficult to prevent and cure because it is associated with high levels of IL-6, which in turn induce the deregulation of the main proteins involved in iron homeostasis: hepcidin, Fpn, Tf, TfRs, Cp and Heph [26]. Therefore AI, characterized by hypoferremia in the blood despite suitable iron stores [27], is very difficult to cure in the absence of a contemporary decreasing of serum IL-6 levels. Conversely, AI has been considered, for a long time, as a host defense mechanism against extracellular pathogens, limiting iron availability in the blood [26,27,28]. This concept should be deeply reviewed in light of the capability of some bacteria to enter and survive inside cells, such as macrophages. Intracellular iron retention could be an inducer of the growth of facultative and obligate intracellular pathogens inside epithelial cells and macrophages, thus increasing infection severity [29,30]. In this respect, the recent discovery of the tight correlation between iron and inflammatory homeostasis must take into account that infectious processes by intracellular bacteria are favored and enhanced by intracellular iron overload, making imperative a strong revision of the classical iron therapy.

It is therefore of utmost importance to counteract the persistence of the inflammatory status to rebalance iron levels between tissues/secretions and blood, thus avoiding intracellular iron accumulation and the increased infection severity.

## 2. Lactoferrin

As already reported, sophisticated strategies have been also developed to bind iron in a nontoxic form, and among these, lactoferrin (Lf) and transferrin (Tf), two iron-binding glycoproteins, exert a pivotal role. Lf, identified in 1939 in bovine milk and isolated in 1960 from both human [31,32] and bovine milk [33], is the most important owing to its multifunctional activities. Human lactoferrin (hLf), a glycoprotein of 691 amino acids, is constitutively expressed and secreted by glandular epithelial cells and by neutrophils following induction. Human colostrum shows the highest levels of Lf (~7 g/L) [34], while mature milk, other secretions and secondary granules of neutrophils present lower levels (Table 1) [35,36]. During infection and/or inflammation processes, the Lf concentration increases through the recruitment of neutrophils. Remarkably, 10^6^ neutrophils synthesize 15 μg of Lf.

**Figure 1:**
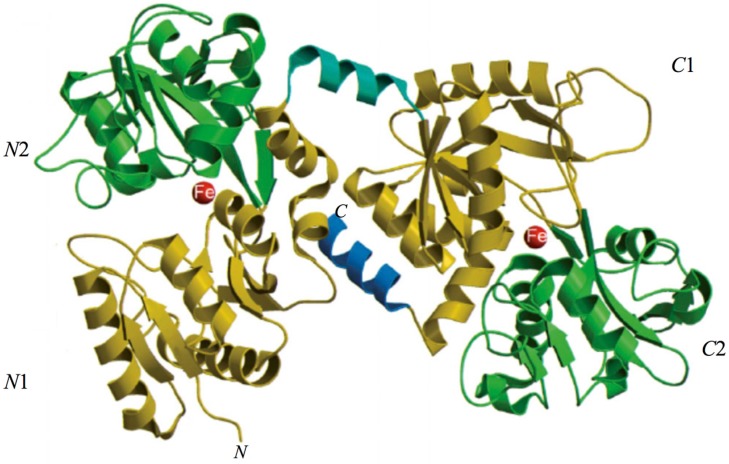
Structure of lactoferrin. The *N*-lobe on the left and the *C*-lobe on the right are divided into four domains, labeled *N*1, *N*2, *C*1, *C*2. The red spheres represent the two ferric ions in each iron-binding site.

The 3D structure of hLf has been described in detail by several authors [37,38,39]. In particular, hLf is divided into two homologous lobes (*N*-lobe residues 1–333 and *C*-lobe residues 345–691) connected by a 3-turn α-helix peptide (residues 334–344) [37,40]. Each lobe, constituted by two domains (*N*1 and *N*2, *C*1 and *C*2), binds one ferric ion and one carbonate anion within a deep cleft between the domains of each lobe [37] (Figure 1). The Fe(III) ligands, highly conserved among most iron-binding proteins [41,42], are identical in both lobes: one aspartic acid, two tyrosines, one histidine (Asp-60, Tyr-92, Tyr-192 and His-253 in the *N*-lobe and Asp-395, Tyr-433, Tyr-526 and His-595 in the *C*-lobe).

**Figure 2:**
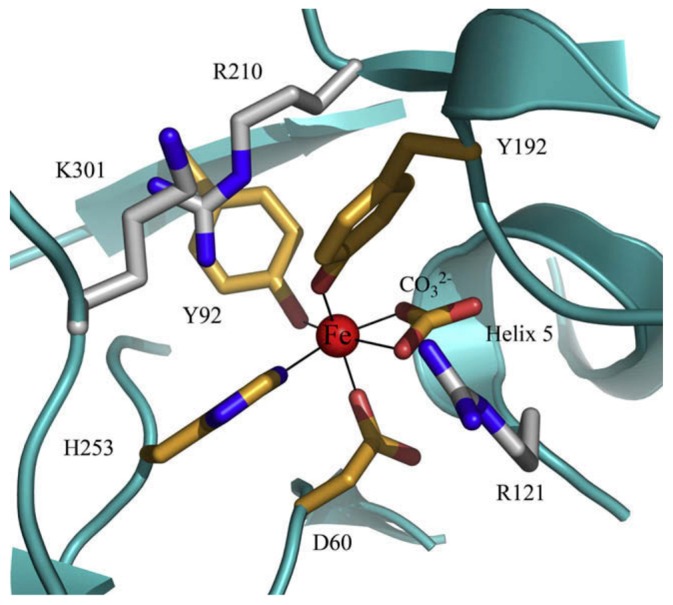
Lactoferrin iron-binding site. Iron-binding site in the *N*-lobe: two tyrosine (Y92 and Y192), one aspartic acid (D60), one histidine (H253) and one carbonate anion together with the arginine residue (R121). Two basic residues behind the iron site, an arginine (R210) and a lysine (K301) help modulate iron release.

**Figure 3:**
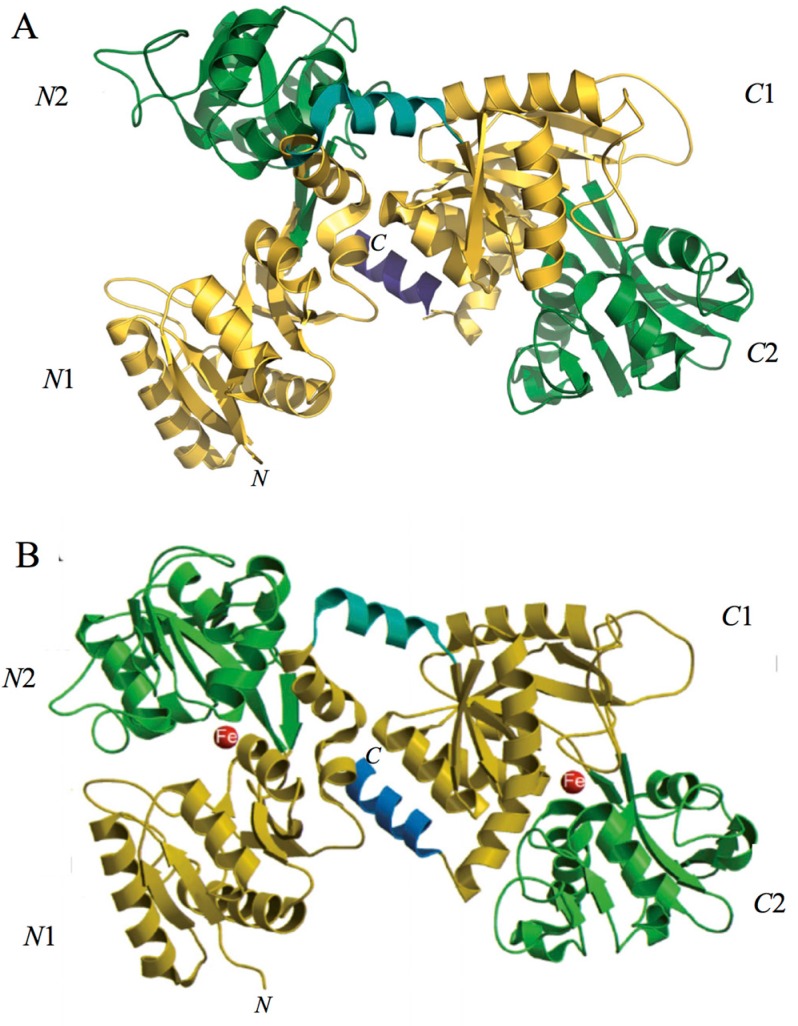
(**A**) Structure of lactoferrin in apo-form (iron-free); and (**B**) structure of lactoferrin in holo-form (iron-saturated).

Spectroscopic studies and the 3D structure suggest that the CO_3_^2−^ anion binds first, thus neutralizing the positive charge of the arginine residue (Arg-121 in the *N*-lobe and Arg-465 in the *C*-lobe) [43,44]. The participation of the CO_3_^2−^ anion in the iron coordination binding appears to be ideal for iron reversible binding [43] since the protonation of the CO_3_^2−^ anion is a likely first step in the breakup of the iron site at low pH [45] (Figure 2). HLf shows a high similarity with other Lfs isolated from bovine [46], horse [47] and buffalo [48]. All Lfs can adopt two main conformational states: the open metal-free (apo-lactoferrin) and the closed metal-bound (holo-lactoferrin). Metal binding and release are thus associated with large-scale conformational changes in which the domains close over the bound metal ion or open to release it [49,50] (Figure 3). The iron-saturated closed form is highly stable and more resistant to digestion by proteases compared to the unsaturated open one [51]. Within the iron-binding transferrin family, Lf is able to reversibly chelate two Fe(III) ions per molecule with high affinity (*K*_d_ ~ 10 ^−20^ M), as well as retain ferric iron until pH values as low as 3.0, characteristic of the infection and inflammation sites. Conversely, Tf retains iron until a pH of about 5.5 [43,52]. Of note, the iron binding ability of Lf in secretions and of Tf in cells and circulation guarantees that free available iron does not exceed 10^−18^ M, thus preventing (i) iron precipitation as insoluble hydroxides, (ii) microbial growth and (iii) the formation of ROS, responsible for tissue, cell, DNA, protein and membrane lipid damage. In addition to the iron-binding ability of Lf and Tf, the closed forms can include other transition metal ions such as Cu^2+^ and Mn^3+^, chelated at lower affinity than Fe^3+^ without changing the basic structure [53,54,55,56,57]. Conversely, the differences in iron-releasing ability are related to their different functions: anti-microbial, anti-inflammatory and immunomodulatory activities for Lf and iron delivery activity for Tf.

### 2.1. Lactoferrin Functions

hLf and bLf possess high sequence homology and exert identical multifunctions: antibacterial, antifungal, antiviral and antiparasitic, anti-inflammatory and immunomodulatory activities [58,59,60]. Therefore, the majority of the in vitro and in vivo studies have been carried out using bLf, generally recognized as a safe substance (GRAS) by the Food and Drug Administration (FDA, USA) and available in large quantities. All of the functions ascribed to Lf can be dependent or independent of Lf-iron-binding ability.

#### 2.1.1. Antibacterial and Anti-Biofilm Activity Dependent on Lf Iron-Binding Ability

**Figure 4:**
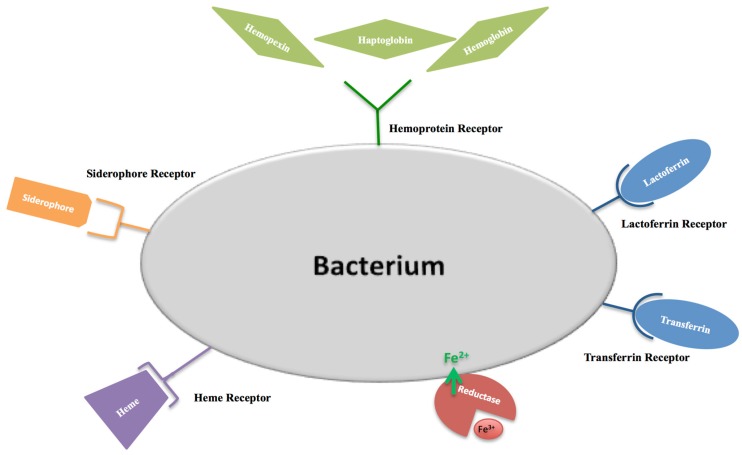
The bacterial iron transport mechanisms: (i) synthesis of high affinity ferric ion chelators, siderophores; (ii) receptor mediated endocytosis of the main iron-binding molecules (lactoferrin, transferrin, hemopexin, haptoglobin, hemoglobin and heme); (iii) passive transport mediated by bacterial reductase.

The first function attributed to hLf/bLf (Lf) was the antimicrobial activity. The bacteriostatic action of Lf is usually iron dependent, as iron supplementation reverts its effect [61]. The Lf antibacterial activity is counteracted by three main mechanisms put in place by bacterial pathogens: (i) synthesis of high affinity ferric ion chelators, named siderophores, that compete with iron-binding proteins for iron acquisition and delivery into bacteria through specific receptors [62]; (ii) iron acquisition through Lf or Tf binding mediated by their specific surface receptors [63,64]; iron acquisition through hemoglobin, haptoglobin and hemopexin binding mediated by surface hemoprotein receptors [65]; iron acquisition through heme binding mediated by the surface hemophore receptor [66]; (iii) iron acquisition through bacterial reductase able to reduce ferric to ferrous ions, thus eliminating the substrate of the Fenton reaction and assimilating ferrous ions that passively enter inside microbial cells [67]. The bacterial iron transport mechanisms are summarized in Figure 4. Later, in 2002, Singh et al. [68] demonstrated another important iron-dependent Lf function: inhibition of *Pseudomonas aeruginosa* biofilm formation in cystic fibrosis (CF) by the iron-binding activity of Lf [68]. As a matter of fact, CF is associated with alterations in the influx and efflux of chloride and sodium ions, which involves also abnormal high concentrations of iron and ferritin in sputum [69]. This increased availability of iron (median value of 6.3 × 10^−5^ M) induces the generation of ROS, which contributes to lung disorders, as well as to the enhanced growth and colonization of *P. aeruginosa* and *Burkholderia cepacia*, two motile Gram-negative pathogens that are a major source of the morbidity and mortality of CF patients. For both bacteria, biofilm formation is one of the major virulence factors. Peptides and proteins of natural non-immune defenses, including Lf, play a crucial role in combating such infections. A striking Singh et al. [68] discovery was that apo-Lf, by chelating iron, inhibits *P. aeruginosa* adhesion and biofilm formation through activation of a specialized form of motility, named switching. Like *P. aeruginosa*, also free-living forms of *B. cepacia* show a noticeable motility under iron-limiting conditions. On the other hand, iron availability or the addition of iron-saturated bLf inhibits the motility and induces abundant *P. aeruginosa* and *B. cepacia* growth and aggregates, evolving into biofilm [70]. In CF patients, however, these protective effects of Lf are compromised by the presence of high iron concentrations and, consequently, by high levels of holo-Lf [71]. Even if the hLf concentration increases in infection and inflammation processes, in sputum of CF patients, free iron concentrations remain higher than in normal subjects [72]. The high iron concentration (6.3 × 10^−5^ M) saturates hLf (1 × 10^−5^ M), thus preventing hLf from inhibiting biofilm formation.

#### 2.1.2. Antibacterial Activity Independent of Lf Iron-Binding Ability

**Figure 5:**
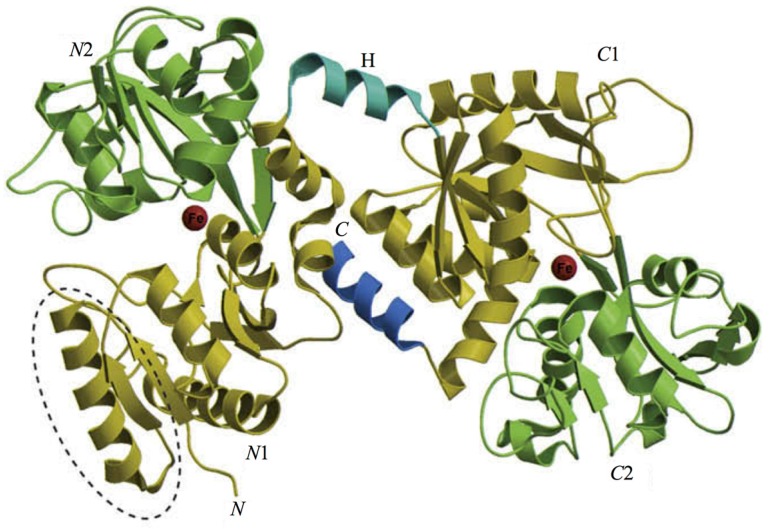
Lactoferricin localization in bovine lactoferrin. The dotted section indicates the bovine lactoferricin.

An iron-independent bactericidal action is exerted by Lf direct interaction with the lipopolysaccharide (LPS) of Gram-negative or with the lipoteichoic acid of Gram-positive bacteria [73,74]. The bactericidal activity of Lf is located in the *N*-terminal region (Figure 5), as its derivative cationic peptide, generated by pepsin digestion, called lactoferricin (Lfcin), is several folds more active than the intact protein in interacting with LPS and in killing Gram-negative bacteria [75,76]. It is also important to underline that the presence of high calcium concentrations can counteract the release of LPS from Gram-negative bacteria induced by Lf. In fact, the ability of Lf to bind Ca^2+^ through the carboxylate groups of the sialic acid residues present on glycan chains provokes the release of significant amounts of LPS from Gram-negative bacteria, without needing a direct interaction with bacteria [77]. The bactericidal activity towards Gram-positive bacteria appears to be related to the same cationic residues involved in the bactericidal activity against Gram-negative bacteria [58].

#### 2.1.3. Inhibition of Bacterial Adhesion on Abiotic and Cell Surfaces

Independently from its iron-binding ability, bLf inhibits the bacterial adhesion to host cells through its competitive binding to host cells and/or to microbial surface components [30,58]. Microbial adhesion and subsequent colonization, resulting in biofilm formation on abiotic surfaces, such as catheters, prosthesis and medical devices, represent a serious problem that can lead to illness and death. Efforts to reduce microbial adhesion, using new materials or compounds inhibiting microbial adhesion, have had modest success once applied to the patient. Consequently, it would be very helpful to discover other compounds able to hinder microbial adhesion. In 1989, the ability of Lf, in both apo- and holo-form, to inhibit the adhesion of *Streptococcus mutans* to hydroxyapatite (HA), mimicking the tooth surface, was an interesting disclosure [78]. The further demonstration that Lf inhibits the adhesion of *S. mutans* to HA through residues 473–538 of its *C*-lobe confirmed that this activity is unrelated to Lf iron-binding properties [79]. The influence of Lf on bacterial adhesion on contact lenses has been also shown through the much lower number of adherent *P. aeruginosa* on hLf-coated lenses compared to that observed on hLf non-coated ones [80]. The different nature of abiotic surfaces, microbial adhesion mechanisms and in vitro experimental conditions indicate that the inhibition of bacterial adhesion by apo- or holo-Lf can explain the different requirement to exert adhesion: ionic binding to biomaterials, as well as specific binding to bacterial structures, or both.

The ability of microbes to adhere, colonize and form biofilm on host cells is also a crucial step in the development and persistence of infections. The first demonstration of the mucosal protective activity of hLf against injury by adherent *Escherichia coli* HB101 was included in the data reported by Longhi et al. [81].

Later, it was confirmed that Lf can inhibit the first step for bacterial pathogenesis through the inhibition of bacterial adherence to host cells [70,82,83,84,85,86,87]. Lf has also been shown to inhibit the adherence of enterotoxigenic *E. coli* (ETEC) to human epithelial cells and to intestinal mucosa of germfree mice [82], as well as the adhesion of three adhesive diarrheagenic *E. coli* strains (DAEC), enteroaggregative *E. coli* (EAEC) [88] and enteropathogenic *E. coli* (EPEC) [83].

hLf and bLf, human Lfcin (hLfcin) and bovine Lfcin (bLfcin) are all able to bind to Gram-negative and Gram-positive bacterial surfaces [89], as well as to host cells, by binding to glycosaminoglycans (GAGs) [90] and specifically to heparan sulfate (HS) [91].

However, Lf can prevent adhesion through other mechanisms. The importance of the sugar residues on Lf is suggested by the observation that whereas native hLf inhibits *Shigella* spp. adhesion [92], recombinant hLf (rhLf), with different glycosylation, has no effect on *Shigella flexneri* adhesion to epithelial cells [93]. Another paper suggests that hLf, rhLf and bLf inhibit the attachment of *Helicobacter felix* to gastric epithelial cells, probably by interaction between oligomannoside-type glycans of Lf and bacterial adhesins that recognize these residues [94]. Although inhibition of bacterial adhesion seems generally to be mediated by Lf binding to both bacterial and host cell surfaces, the surprising discovery of hLf proteolytic activity [95] provided an additional mechanism to explain Lf anti-adhesive activity. Thus, inhibition by hLf of the adhesion of EPEC strains [83], which use a type III secretory system to deliver effector proteins into the host cell, was ascribed to hLf-mediated degradation of the secreted proteins, EspA, B, D [96], as well as hLf inhibition of *H. influenzae* and *Aggregatibacter actinomycetemcomitans* adhesion to the degradation of two colonization factors and of autotransporter proteins, respectively [97,98,99].

Although the experimental conditions of the studies reported were different, the pre-incubation of Lf with host cells seems never to inhibit Gram-positive and Gram-negative bacterial adhesion, suggesting that Lf binding to GAGs or HS is not crucial. Instead, the inhibition of Gram-positive and Gram- negative bacterial adhesion by Lf seems to require Lf binding to bacteria or a putative Lf-mediated degradation of the adhesins or proteins of the secretory systems. During the adhesion process, bacteria are unable to stimulate the epithelial cell inflammatory responses at significant levels [100].

#### 2.1.4. Inhibition of Bacterial Entry into Host Cells

Some mucosal pathogenic bacteria are capable not only of adhering, but also of entering into non-professional phagocytes, such as epithelial cells. Inside host cells, bacteria are in a protective niche in which they can replicate and persist, thus avoiding host defenses. In addition, antibiotic therapies are not always effective at eradicating intracellular pathogens [101]. Virulence determinants, such as surface proteins able to bind host cells, play a key role in the entry process inside the host cells. Lf has been shown to inhibit the entry of Gram-negative and Gram-positive facultative intracellular bacteria. The first demonstration of the inhibition of bacterial invasion by bLf involved *E. coli* HB101(pRI203), a recombinant strain able to enter inside cells owing to the *inv* gene located in pRI203 plasmid [81]. Bacterial entry into host cells is mediated by the binding of bacterial invasin, a product of the *inv* gene, to the host integrin receptor. The effectiveness of apo- and holo-bLf and bLfcin towards *E. coli* strain HB101 (pRI203) invasion is correlated with their ability to bind to both cultured cells and the bacterial outer membrane [81,102]. Likewise, for *Y. enterocolitica* and *Y. pseudotuberculosis*, grown in conditions allowing maximal invasin synthesis, a 10-fold inhibition of invasion of cultured cells by bLfcin was observed [102]. It appears that the binding of Lf and Lfcin to integrins through the same domains that are targeted by invasin, and to GAGs and/or HP, can induce a dramatic subversion in bacterial-host cell interaction, thus inhibiting bacterial internalization [84]. Similar mechanisms apply to the inhibition of the invasion of the Gram-positive bacteria *L. monocytogenes*, *Streptococcus pyogenes* (GAS) and *Staphylococcus aureus*, i.e., apo- or holo-bLf binding to both bacterial adhesins and host cells [29,103,104]. The ability of bLf to decrease GAS invasion was also confirmed by an in vivo trial carried out on 12 children suffering from pharyngitis and already scheduled for tonsillectomy [29]. Although all studies, reported above, have been carried out with different facultative intracellular microorganisms in different in vitro models, Lf, in apo- or holo-form, always exerts an inhibiting activity against the microbial internalization [58,85,105]. In contrast to inhibition of bacterial adhesion, Lf binding to GAGs and/or HP of host cells seems crucial in inhibiting bacterial internalization. In the invasion process, the facultative intracellular bacteria induce the up-expression of pro-inflammatory cytokines by infected epithelial cells [87,100,106]. BLf can efficiently inhibit the invasion of an obligate intracellular bacteria, as *C. trachomatis*, as well as counteract the inflammatory process promoted by its intracellular localization [30].

*C. trachomatis*, responsible for the most common sexually-transmitted bacterial disease worldwide, is asymptomatic in about 80% of women and causes acute and chronic infections. Unlike acute infections, which can be cured with antibiotics, chronic infections are difficult to eradicate and need prolonged therapies, thus increasing the risk of developing antibiotic resistance [107]. Even if novel alternative therapies are needed, the difficulty in finding new agents against *C. trachomatis* resides in the complex biphasic developmental life-cycle of this peculiar pathogen: extracellular infectious bodies (elementary bodies, EBs) metabolically inactive, and the intracellular non-infectious bodies (reticulate bodies, RBs), metabolically active. In addition, *C. trachomatis* infection induces inflammatory processes. The up-expression of pro-inflammatory cytokines such as TNF-α, IL-1β, IL-6 and IL-8 induces direct damage to genital tissues. A great interest in Lf, considered as a prominent component of the first-line defense of the host against infections and inflammation, has been raised. Recently, the protective effect of bLf against *C. trachomatis* infection and inflammation in vitro and in vivo has been demonstrated. A preparation of bLf, iron-saturated at 20%, to consent to further iron chelation, was utilized in in vitro model to check its anti-chlamydial activity [30]. The incubation of cell monolayers with bLf before the infection or at the moment of the infection significantly inhibited the adhesion and entry of elementary bodies of *C. trachomatis* into epithelial cells. Therefore, the inhibition of *C. trachomatis* infectivity by bLf was dependent on its interaction with the cell surface and especially with GAGs and HS proteoglycans [90,108], which are potential receptors for *C. trachomatis* adhesion [109]. Conversely, the preincubation of bLf with *C. trachomatis* EBs did not influence its infectivity, supporting the idea that the specific interaction between bLf and epithelial host cells could be the sole pivotal mechanism responsible for the inhibition of *C. trachomatis* invasion [30]. Interestingly, the addition of bLf significantly decreased the IL-8 and IL-6 levels synthetized by *C. trachomatis*-infected cells. These results, demonstrating once again the ability of bLf to down-regulate pro-inflammatory cytokine synthesis and showing for the first time the protective effects of bLf against *C. trachomatis* infection, led us to investigate its efficacy also in asymptomatic pregnant women positive for *C. trachomatis* and with high levels of IL-6 in cervical fluids. In a pilot study, seven out of 176 pregnant women enrolled, showing cervical specimens positive for *C. trachomatis*, were treated with the intravaginal administration of bLf (100 mg) every 8 h for 30 days. Interestingly, after one month, six out of seven pregnant women were negative for *C. trachomatis* and showed significant decreased IL-6 levels in their cervical vaginal fluids [30]. Similar to what was observed in the in vitro model, intravaginal administration of bLf seems to act by protecting host cells against the adhesion and entry of chlamydial EBs, which are released extracellularly after redifferentiation of RBs to EBs. The simultaneous decrease of IL-6 levels could be a marker for the lack of *C. trachomatis* EBs infection of host cells due to the presence of bLf. In other words, bLf protects host cells, preventing the early phase of infection by EBs. Therefore, the in vivo anti-chlamydial activity of bLf is related to the protection of host cells against the adhesion to and entry into host cells of *C. trachomatis* EBs, as well as to its anti-inflammatory function [30].

#### 2.1.5. Inhibition of Viral Infections

The antiviral activity of hLf was described, for the first time, in mice infected with the polycythemia-inducing strain of the Friend virus complex [110]. Since 1994, an effective antiviral activity of both hLf and bLf during the early stage of infection of enveloped and naked viruses was demonstrated. This activity is mainly due to bLf binding to GAGs and HS or viral particles or both ([111] and references therein), thus inhibiting viral entry into host cells. Despite the antiviral effect of Lf widely demonstrated in in vitro studies, few clinical trials have been carried out, and the related mechanism of action is still under debate.

Nevertheless, the ability of Lf to exert a potent antiviral activity strengthens the idea that this natural glycoprotein is an important brick in the mucosal wall, effective against viral attacks, and it could be usefully applied as a novel strategy for the treatment of viral infections and of inflammation, the major contributing factor to viral disease severity [112]. Epidemiological evidence and clinical observations of infections in humans suggest that different viruses may be associated with different inflammatory responses. Whether or not these differences can be attributed to the viruses themselves or to hosts that are susceptible to severe infection or prone to produce high levels of inflammation with a given virus is still under debate.

#### 2.1.6. Anti-Inflammatory Activity of Lf in Infected and Inflamed Host Cells

As already reported, Lf possesses a potent anti-inflammatory activity able to both modulate the inflammatory response by epithelial cells infected by facultative and obligate intracellular bacteria [30,87,100,106] and revert/attenuate the inflammatory response triggered by Toll-like receptor engagement in antigen-presenting cells [59,60].

Nevertheless, the literature is full of papers showing contradictory effects of Lf on inflammatory processes in different in vitro cell models. However, a deeper analysis of these conflicting in vitro models revealed how experimental conditions can affect the results. In particular, the experimental cell line models, such as epithelial or phagocytic cells, the different type of infecting agents, such as bacteria or viruses or the stimulation with pathogen-associated molecular patterns (PAMPS), such as flagellin, toxin, peptidoglycan, lipopolysaccharide (LPS), and, finally, the use of Lf from different sources are all important factors that can influence the results [113,114,115,116]. It is very important to underline the different inflammatory response by epithelial or macrophagic cells injured by the same stimulus. Epithelial cells are less responsive to bacterial PAMPS, such as LPS, compared to the high responsivity of phagocytes [4,5,87,117,118]. In particular, cultured epithelial cells treated with LPS or infected by non-invasive adherent *E. coli* HB101 synthesize very low levels of pro-inflammatory cytokines, making it difficult to highlight the anti-inflammatory activity of bLf [87,100]. Conversely, the same monolayers infected by invasive *E. coli* HB101 (pRI203) significantly up-express pro-inflammatory cytokines, the synthesis of which is significantly decreased by bLf [100]. These two isogenic *E. coli* strains express an identical LPS, but *E. coli* HB101 is only able to adhere to surface cell structures, similarly to commensal bacteria, while *E. coli* HB101 (pRI203) is capable of entering the host through cells as intracellular pathogenic bacteria. Therefore, the different levels of pro-inflammatory cytokines synthesized by infected epithelial cells are independent of the LPS structure, but strongly dependent on the localization of viable infecting bacteria: adherent or intracellular. It is important to underline that the anti-inflammatory activity of bLf was tested in vitro in different epithelial monolayers untreated or treated with bLf at a concentration <100 µg/mL, which does not inhibit the entry of facultative or obligate intracellular bacterial pathogens. Consequently, the monolayers that were untreated or bLf-treated contain a similar number of intracellular bacteria. If the experiments were not designed as described, the anti-inflammatory activity of bLf would be incorrectly ascribed to the different numbers of intracellular bacteria.

Different epithelial monolayers infected with various facultative or obligate intracellular pathogens were found to up-express pro-inflammatory cytokines. The addition of bLf at 100 µg/mL significantly decreased IL-1β, IL-6, IL-8 and NF-κB levels [30,87,100,106]. BLf also exerts its anti-inflammatory activity in LPS-inflamed macrophages [4,5]. Human macrophages, responsive to LPS treatment, up-express IL-6, which is significantly inhibited by bLf, which reduces the pathological inflammation and cell damage, similarly to what was observed in epithelial cells invaded by intracellular bacteria [4,5,87].

Of note, the anti-inflammatory activity of Lf had been firstly hypothesized by the demonstration that exogenous bLf is internalized from the apical side of host cells and localized in the nuclei [119]. In agreement with the nuclear localization of Lf, in 2008, a very elegant and important paper by Suzuki et al. [120] revealed that the *N*-lobe or the *N*1.1 sub-domain of Lf is sufficient for binding, internalization and targeting to the nucleus of host cells. The capacity of bLf to reach the nucleus has been shown in different cell monolayers, including intestinal cells [121], and in freshly-isolated monocytes [60]. The bLf ability to localize into the nuclei of these cells is comparable to that showed by hLf in endothelial cells [122]. Consequently, the nuclear localization strongly suggested that this molecule may be involved in the transcriptional regulation of some genes of host inflammatory responses, thus acting as a transcriptional factor and modulator of the inflammatory processes through the inhibition of pro-inflammatory cytokines [60,106,121,122].

## 3. Lf and Anemia of Inflammation

Although the mechanisms by which bLf exerts its anti-inflammatory activity are under debate, in 2006, by designing the first clinical trial on the effect of 30 days of bLf oral administration (100 mg two times a day before meals) in pregnant women with IDA or AI, we obtained surprising results [19]. In fact, pregnant women receiving 100 mg of bLf, iron saturated at 20–30% two times a day acquired 70–84 µg/day of iron, respectively. Although the concentration of iron supplemented by bLf is very far from that which is required daily (1–2 mg), a significant increase of the concentration of Hb and TSI was detected after 30 days of the treatment. Therefore, we speculated that bLf efficacy in curing AI was presumably not linked to direct iron supplementation, but to a more complex mechanism involving this protein in iron homeostasis. Later on, in other clinical trials, bLf treatment showed a significant improvement of hematological parameters, including red blood cell number, hemoglobin, total serum iron, serum ferritin concentrations and percentage of hematocrit, in pregnant women suffering from IDA, associated with a consistent decrease of serum IL-6 levels [121,123] (Table 2). Lf is, therefore, a key element, not only in the host defense system [58,124,125], but also a pivotal component able to inhibit the inflammatory response, especially in inflamed pregnant women affected by hereditary thrombophilia [126] (Table 3).

In all of these clinical trials, oral administration of bLf to IDA or AI pregnant women was compared to the classical therapy with ferrous sulfate [19,123,126,127]. We have demonstrated that these pregnant women did not adequately respond to oral iron administration [19,124,128]. Surprisingly, pregnant women treated with oral ferrous sulfate showed an increase of serum IL-6 with a contemporary failure of the increase of hematological parameters [123,129] (Table 4). Oral ferrous sulfate was also demonstrated to be ineffective against anemic non-pregnant women [129] and in other subjects, including hemodialysis patients [130].

Iron supplementation in AI patients could heighten iron overload in tissues and secretions, thus increasing both susceptibility and severity of infections, especially in developing countries with a high incidence of microbial and parasitic infections [131]. The proof that iron administration is unable to decrease any type of inflammatory process should not be surprising, for iron is itself an enhancer of inflammation [127,128,129].

In this respect, the novel and hopeful approach to treat AI with bLf in place of iron supplementation is of utmost importance. In recent studies, carried out in Italy on more than a thousand pregnant women suffering from ID or IDA or AI, oral bLf treatment was proven to be safe, without any side effects, and more effective than the classical ferrous sulfate therapy in both rebalancing hematological parameters and, overall, decreasing serum IL-6 levels (Table 2, Table 3 and Table 4). A reasonable explanation of the mechanism of bLf against iron and inflammatory disorders was found in the recent experiments carried out on inflamed and uninflamed human macrophages in the presence or absence of bLf [4,5]. The results obtained in LPS-inflamed macrophages versus uninflamed ones, in the absence of bLf, showed high levels of IL-6 associated with a decrease of Fpn, TfR and Cp, as well as an increase of intracellular Ftn and iron concentration. Inflamed macrophages were iron overloaded and down-expressed Fpn. This behavior in vivo is associated with anemia of inflammation (AI) [18,121]. Of note, in inflamed macrophages, the addition of bLf restored Fpn, Cp and TfR synthesis and decreased intracellular Ftn [5]. The capacity of bLf to reduce pro-inflammatory cytokines production and to prevent the changes of the whole set of proteins involved in iron homeostasis, in inflamed macrophages, underlines the pivotal role of this natural compound in the complex orchestration of iron and inflammatory homeostasis.

Although the discovery of the Fpn-hepcidin complex has greatly helped to define the sophisticated iron homeostasis mechanisms, the preconception that considers oral or intravenous iron administration as a logical intervention to increase hemoglobin concentration and reduce the incidence of AI is still prevalent despite the limited, if not the harmful, effects of iron supplementations. The reduction of circulating iron can be associated with dangerous iron load or overload in cells. These changes in iron status can thus affect microbial growth and the severity of infections especially in the areas of the world where infections such as malaria and tuberculosis are highly prevalent, contributing to the high prevalence of severe anemia, morbidity and mortality. Therefore, iron supplementation in these areas is not only futile and ineffective in increasing hematological parameters, but also potentially harmful, because, during the acute phase of infections, the increased IL-6 levels enhance the hepcidin and down-regulate Fpn, thus increasing the risk of more severe infections.

We strongly believe that in vivo, the actual condition of anemia of inflammation consists of iron delocalization, i.e., iron overload in cells and tissues and iron deficiency in blood. Consequently, the deficiency of iron must not be considered as a lack, but as a delocalization of iron.

In this respect, the surprising results obtained for subjects affected by ID, IDA or AI treated with bLf oral administration clearly demonstrated that bLf restores iron localization through the decrease of IL-6 which in turn, decreases iron intracellular overload due to new Fpn up-expression.

Recently, bLf was found to induce the shift from inflammatory macrophagic M1 to tolerogenic M2 phenotypes [5]. Several drugs capable of modulating macrophagic phenotypes are emerging as attractive molecules for treating AI, and in this sense, bLf is no exception.

## 4. Anti-Inflammatory Activity of bLf in Preventing Preterm Delivery

These new Lf functions, effective I n curing ID, IDA and AI [121,123], as well as in restoring iron and inflammatory homeostasis, could have great relevance not only in developed but also in developing countries, where iron deficiency and inflammation-associated anemia represent the major risk factors of preterm delivery (PTD) and maternal and neonatal death [121]. In pregnancies, both cervical and vaginal infections [132,133], as well as cervicovaginal sterile inflammations, could increase the threat of PTD [134]. Contradictory data on the link between maternal serum IL-6 levels and PTD are reported [135,136,137,138]. Conversely, IL-6 concentration in cervicovaginal and amniotic fluids seems tightly related to PTD, indicating that inflammatory processes at the maternal-fetal interface, rather than systemic inflammation, play a pivotal role in PTDs [138]. As a matter of fact, IL-6 stimulates the expression of prostaglandin F2a (PGF2a), a major inducer of uterine contractions and premature membrane ruptures [139]. This coordinated interplay between IL-6 and PGF2a regulates both preterm and term delivery [140]. In this respect, anti-inflammatory molecules, such as prostaglandin synthase inhibitors, inhibit uterine activity, thus extending pregnancy [141]. Although progress has been made in outlining some factors involved in PTD, more insights are needed to identify PTD complex regulatory circuits. In particular, the relationships between PTD and iron and inflammatory homeostasis disorders remain to be proven. An open-label cohort and subcohort study on 161 anemic pregnant women, designed to confirm the previous studies on the effect of bLf oral administration on iron and inflammatory homeostasis in ID/IDA pregnant women, demonstrated how combined oral and intravaginal bLf administration on 15 anemic pregnant women with sterile PTD threat significantly rescued hematological parameters, as well as IL-6 levels in both serum and cervicovaginal fluid, thus extending the pregnancy length. The intravaginal administration consisted of 100 mg of bLf every 8 h for at least four weeks and in any case no longer than the 37th gestation week. The efficacy of bLf therapy was evaluated by ultrasonographic measurement of the cervical length and by quantitation of IL-6, PGF2a and fetal fibronectin (fFN) levels in cervicovaginal fluids. In particular, among 15 women with the perception of PTD threat: (i) four were tangibly found to not be at PTD risk, as detected by a cervical length > 30 mm and by mean values of fFN < 50 ng/mL, IL-6 < 120 pg/mL and PGF2a < 50 ng/mL in cervicovaginal fluid; (ii) 11 women at PTD risk without premature rupture of membranes, showing a cervical length < 30 mm and mean values of IL-6 >120 pg/mL, PGF2a > 50 ng/mL and fFN < 50 pg/mL in cervicovaginal fluid. After bLf intravaginal administration, uterine contractions were thought to fade, contemporary with the actual decreasing of both IL-6 and PGF2a concentrations in cervicovaginal fluid. Moreover, bLf intravaginal administration, without any side effect, blocked both increasing of fFN, as well as shortening of cervical length, thus extending the pregnancy [121].

In another recent clinical trial, seven asymptomatic pregnant women, positive for *C. trachomatis* and with a high concentration of IL-6 in cervical fluids, were treated with bLf intravaginal administration (100 mg every 8 h for 30 days) to inhibit *C. trachomatis* infection, as well as to decrease IL-6 in cervical fluids, thus diminishing the threat of putative PTD [30]. After one month of bLf treatment, six cervical specimens were negative for *C. trachomatis*, with a contemporary decreasing in IL-6 levels in the cervical fluids (from mean values of 250 down to 50 pg/mL). Only one pregnant woman remained positive for *C. trachomatis* with still a high cervical IL-6 concentration (about 270–300 pg/mL). No adverse effects following bLf intravaginal administration were observed in pregnant women [30].

## 5. Lf in Oral Pathologies: Surprising Poor Oral Health Related to Iron and Inflammatory Homeostasis Disorders in Athletes Participating in the Olympic Games

Gingivitis is the most common oral disease associated with plaque accumulation in the gingival-dental area. In the early phase, the disease is confined to gingiva, and no dental attachment loss is observed [142]. Later on, the supporting structures become involved, and the term ‘marginal periodontitis’ is used to describe the disease, which leads to gingival swelling, bleeding and bad breath. In the late phase of the disease, the supporting collagen of the periodontium is degenerated, the alveolar bone begins to resorb, and gingival epithelium migrates along the tooth surface forming a “periodontal pocket” [143,144,145]. The periodontal pocket provides ideal conditions for the proliferation (primarily) of Gram-negative anaerobic facultative intracellular bacteria, such as *Porphyromonas gingivalis*, *Aggregatibacter actinomycetemcomitans* and *Prevotella* spp. In particular, *P. gingivalis*, the most frequently-isolated bacterium in patients affected by periodontitis, exhibits an obligate requirement for heme/hemin, so that its growth is favored by the bleeding. Periodontitis affects roughly half of the world population over the age of 45 [146]. Periodontal disease has been considered as a possible risk factor for other systemic diseases such as cardiovascular diseases and preterm low birth weight infants [147]. Periodontitis requires extensive treatments, whose failure may lead to teeth loss. The clearance of the sub-gingival infection and elimination of the sub-gingival bacterial plaque and periodontal pockets are considered a priority in the treatment of periodontitis (scaling and root planning). The use of systemic antibiotics for its treatment is recommended only in progressing or refractory periodontitis. Indeed, multiple systemic doses of antibiotics have shown several drawbacks including: (i) inadequate antibiotic concentration at the site of the periodontal pocket; (ii) a rapid decline of the plasma antibiotic concentration to sub-therapeutic levels; (iii) development of microbial resistance; and (iv) high peak-plasma antibiotic concentrations, which may be associated with side effects. These disadvantages have evoked novel treatments to prevent and cure oral infections, including periodontal diseases. Recently, it has become clear that periodontitis is a chronic inflammatory and infectious disease characterized by host immune response and periodontopathogenic bacteria surrounded by esopolysaccharides forming biofilm. The peculiarity of periodontitis implies that the host inflammatory destructive response against biofilm is itself the main cause of the severe damage of the periodontium. In fact, gingival epithelial cells in response to bacterial challenge up-express pro-inflammatory cytokines (IL-1β, IL-6, IL-8 and TNF-α) [148], which destroy cell-cell junctions also through the downregulation of E-cadherin, connexin and claudin [149,150,151].

On the other hand, the periodontopathogenic bacteria are predominantly Gram-negative anaerobic facultative intracellular pathogens whose LPS induces pathogenic inflammation contributing to the progression of periodontitis. The resolution of inflammation, an active, well-orchestrated return of tissue homeostasis, is pivotal to cure periodontitis. However, there is an important distinction between the anti-inflammatory process and resolution; the anti-inflammatory process is a pharmacologic intervention in inflammatory pathways, whereas resolution includes biologic pathways restoring homeostasis. Growing evidences suggest that, in order to cure chronic inflammatory periodontal disease, the analysis of the hematological parameters characterizing iron homeostasis disorders, as ID, IDA and AI, as well as the assays to detect the concentrations of salivary pro-inflammatory cytokines must be executed [148]. The resolution of periodontal disease involves the decrease of iron overload in oral tissues and secretions. In physiological conditions, the availability of free iron in tissues and secretions does not exceed 10^−18^ M, while in pathological conditions, the high concentrations of free iron (about 100 µM) induce microbial multiplication, ROS, cell damage and inflammation. A therapeutic strategy, addressed to decrease iron overload, could counteract and inhibit microbial growth and ROS production, thus hindering cell damage and inflammation. In this contest, we have taken into account human saliva containing several proteins of innate immune defense including Lf, surprisingly active in restoring iron homeostasis disorders.

Every day, from 1000 to 1500 mL of saliva, containing a total of 20–50 mg of Lf, are excreted by salivary glands. Salivary Lf counteracts oral pathogen growth, including *Streptococcus* spp., *Candida albicans* and representative anaerobic periodontopathic bacteria, such as *Aggregatibacter actinomycetemcomitans*, *Porphyromonas gingivalis* and *Prevotella intermedia* residing in biofilm lifestyle in supragingival and subgingival plaque, respectively. Interestingly, Lf exhibits not only antibacterial activity against planktonic forms of *P. gingivalis* and *P. intermedia*, but also inhibits biofilm formation of these bacteria at physiological concentrations [68,152]. Of note, biofilm exhibits lower susceptibility than the planktonic lifestyle and a higher resistance to antibiotics due to high recombinant frequency of antibiotic-resistant strains [153,154]. Therefore, the inhibitory effects of Lf on biofilm development, together with the antibacterial ([58] and the references therein), antiadhesive [155] and anti-inflammatory [156] activities may have beneficial effects on the prevention and cure of periodontal diseases [148]. Oral administration of bLf reduces *P. gingivalis* and *P. intermedia* in the subgingival plaque of chronic periodontitis patients, supporting the idea that bLf is a biofilm inhibitor of periodontopathic bacteria in vitro and in vivo [157]. Of note, in our observational preclinical study on 13 volunteers (seven female and six men, age range 42 to 63 years) suffering from mild chronic periodontitis, the orosoluble tablets, containing 50 mg bLf, to be orally taken two times a day after accurate dental hygiene have been efficient in decreasing the plaque index (PlI), the gingival index (GI), probing depth (PD) and bleeding on probing (BOP), as well as in increasing the clinical attachment level (CAL) [148].

Recently, the efficacy of the treatment of the three bLf orosoluble tablets, after accurate oral hygiene, has been tested on another 50 volunteers with mild periodontitis. At each visit, the subjects were clinically evaluated for PlI, GI, PD, CAL, BOP and for IL-6 in gingival crevicular fluids before and after orally-dissolved bovine bLf. The preliminary results on 30 out of 50 volunteers, reported in Table 5, have confirmed the previous results [148] highlighting the significant anti-inflammatory activity of bLf as shown by the decrease of IL-6 in crevicular fluid.

A recent interesting study was performed on the oral health of the athletes participating in the London 2012 Olympic Games, as well-being is required to optimize athletic performance [158]. The aim of this study was to evaluate oral health, the determinants of oral health and the effect of oral health on well-being, related to the training and performance of athletes. The athletes were enrolled the day before the Opening Ceremony, and the clinical trial ended the day after the closing of the Olympic Games. The results demonstrated that the oral health of athletes was poor with a resulting substantial negative impact on well-being, training and performance. Fifty-five percent of athletes showed dental caries, 45% dental erosion, 76% gingivitis and 15% periodontitis. However, the poor level of their oral health was not a novelty. In fact, the earliest reports on athletes participating in previous Olympic Games reported similar oral pathologies [159,160]. Analysis of salivary Lf showed low protein levels during training and performance due to a decreased saliva production [161,162]. The consequent low salivary Lf concentration decreases its protective role against infections and inflammation in the oral cavity. Furthermore, xerostomia (dry mouth), due to low saliva flow, increases the consumption of energy drinks, usually containing carbohydrates, which can promote oral bacteria multiplication, dental plaque and salivary pH lowering, thus increasing the incidence of caries and dental erosive wear [163].

Noteworthy, the putative causes of the poor level of athlete oral health were also identified in the diet [164]. However, even if these clinical trials have been stimulating, in light of this review on the natural substance Lf, it should be underlined that the parameters characterizing the iron and inflammatory homeostasis have been ignored. Just think that in 1985, Taylor et al. [165] reported that, after a prolonged training or an effort in a performance, athletes showed a transitory iron deficiency in blood, low levels of iron-saturated Tf and high levels of C-reactive protein. The iron deficiency and the C-reactive protein up-expressions were attributed to muscle injury. As reported in this review, recently, it has been demonstrated that iron deficiency is due to the more complex mechanism related to inflammatory disorders. The systemic iron homeostasis disorders in athletes should not be unequivocally attributed to diet iron deficiency, unreal and inexplicable in developed countries, but to iron delocalization: iron overload in tissues and secretions and iron deficiency in blood as a consequence of the up-expression of pro-inflammatory cytokines, including IL-6. It has been, recently, reported that during training, athletes show a transitory inflammation due to high levels of IL-6 and other pro-inflammatory cytokines, as well as up-expression of the inflammation-induced hepcidin involved in ID, IDA and AI [166].

It should be also reminded that in athletes, the transitory anemic status must not be treated with oral administration of ferrous iron because it is ineffective against anemia and can increase IL-6 in serum and in the oral cavity [148]. In addition, oral iron administration can also induce ROS, which in turn, cause cell damage. The production of ROS leads to oxidative stress, which increases muscle fatigue and decreases athletic performance.

Recently, in a clinical trial, oral administration of bLf (100 mg before meals) from two to four times a day, depending on the prolonged training or performance, increased hematological parameters and decreased serum IL-6 levels. This trial is still in progress, as well as another clinical trial where preliminary results indicate that the bLf oral administration is efficient in eliminating bacteria associated with halitosis, gingivitis and periodontitis (manuscript in preparation).

## 6. Conclusions

Lactoferrin (Lf), a multifunctional cationic glycoprotein constitutively synthesized by exocrine glands and by neutrophils following infection and inflammation, is present in human fluids. The detection of the amino acid sequence and three-dimensional structure more than 30 years ago established it is an iron-binding protein belonging to the transferrin family. Lf and transferrin (Tf) have similar amino acid compositions, secondary structures (including their disulfide linkages) and tertiary structures, although they differ in terms of biological functions due to Lf ability to retain iron until a pH of about 3.0, a positively-charged surface at physiological pH and other surface features that give Lf additional functional peculiarities. Similarly to more than 50% of eukaryotic proteins, Lf and Tf are glycosylated. The glycans attached to Lf are different, complex and more heterogeneous than those attached to Tf. The heterogeneity of the glycans between Lf and Tf, as well as the complexity of those attached to Lf were believed to be the basis for at least a part of the differences in the respective biological properties [167]. The glycans are specific for each Lf and Tf, natural or recombinant, and for each species [168].

Mammalian glycans are usually involved in multiple cellular mechanisms that are related to health and disease, and Lf-associated glycans are no exception. However, although several years ago the role of glycans had been associated with the different Lf functions in host defense, to date, this statement appears unclear, not fully understood and still under debate [169]. It should be also kept in mind that the in vitro studies on Lf functions are reported to be influenced by several other factors, such as the experimental conditions. The relationship between Lf functions and glycosylation sites should be deepened, especially through the release of glycans from the polypeptide chain, a necessary step to characterize their role. In this respect, a research work demonstrated that the removal of bLf sialic acid enhances the anti-rotavirus activity of this protein [170].

In vitro studies on the different biological functions of Lf must strongly take into account:

- that antimicrobial Lf activity is influenced by the different microbial genera tested;
- that anti-adhesive Lf activity, independent from Lf iron saturation and from its binding to cell GAG and HS, is dependent on the different abiotic or cellular structures studied;
- that anti-invasive Lf activity, independent from Lf iron saturation, but dependent on its binding to cell GAG and HS, is strictly influenced by the different cellular monolayers tested and from the different invasion mechanisms carried out by facultative or obligate intracellular pathogens;
- that antiviral Lf activity, against enveloped or naked viruses, can be or not related to its anti-inflammatory activity;
- that anti-inflammatory Lf activity is dependent on the experimental model chosen.

Concerning this last point, literature data show contradictory effects of Lf on inflammatory processes assayed in different in vitro cell models, thus making it difficult to understand the actual mechanisms through which Lf exerts the anti-inflammatory activity. A deeper analysis of these conflicting results, in different in vitro models, revealed how experimental conditions can affect the results. As herein reported, it is very important to distinguish if the inflammatory response is carried out by the same stimulus on epithelial or macrophagic cells: the epithelial cells are less responsive to bacterial PAMPS, such as LPS, than phagocytes [4,5,87]. In addition, epithelial cell monolayers express significant levels of pro-inflammatory cytokines only when challenged with invasive microorganisms, while no significant levels are detected following adherent bacteria challenge [87,100].

Taking these data together, we strongly believe that the results on the peculiar biological functions of Lf, in well-controlled in vitro models, are similar and entirely ascribable to its iron-binding activity, its cationic feature, as well as to its ability to enter into the nuclei, thus modulating pro-inflammatory cytokines [119,120,121].

Even if the number of in vitro papers on Lf functions is greater than the in vivo ones, recently, the clinical trials have been increasing. However, the clinical trials performed on Lf functions, sometimes, report conflicting data, as well as a different efficacy of Lf in preventing and curing some human pathologies. Therefore, it is, again, of utmost importance to know the characteristics of commercial Lf utilized in each single study, in order to define the quality of Lf and its peptides, required to exert its claimed functions. Commercial Lf digested in different large fragments or with a high percentage of iron saturation (>30%) does not exert the same activity of the undigested pure Lf. The numerous infringements on the functions of natural substances, although protected by the relative patents, have to be prevented. One way to stop these illegal infringements will be discussed during the next XIII International Conference on Lactoferrin, Rome 5 to 10 November 2017, where the experimental procedures to characterize and control the quality of Lf and its peptides, to be commercialized in Lf medical foods or food supplements to prevent and cure human pathologies, will be established.

The most important human pathologies currently under investigation in increasing clinical trials are directed toward Lf activity against infectious diseases by facultative and obligate intracellular pathogens, inflammatory processes, sepsis and necrotizing enterocolitis in preterm infants, anemia of inflammation and oral pathologies, thus suggesting that this multifunctional protein is becoming useful in clinical practice against human diseases.

## Figures and Tables

**Table 1:** Lactoferrin concentration in different human fluids and secretions.

Biological Fluids	Concentration (mg/mL)
Colostrum	8
Milk	1.5–4
Tears	2
Saliva	0.008
Vaginal secretion	0.008
Seminal fluid	0.112
Cerebrospinal fluid	Undetectable
Plasma	0.0004
Joint fluid	0.001

**Table 2:** Hematological values of 869 pregnant women suffering from iron deficiency anemia (IDA) treated for 30 days with 100 mg of bovine lactoferrin (bLf) two times a day before meals. Statistical analysis was performed by ANOVA. * Significant differences (*p* < 0.0001).

Serum Parameters	Mean Values ± Standard Deviation					
	RBC 10^3^/mmc	Hb g/dL	TSI µg/dL	sFtn ng/mL	Hematocrit %	IL-6 pg/mL
Before bLf treatment	3680 ± 216	10.8 ± 0.5	56 ± 22	13 ± 7	29 ± 3	25 ± 7
After bLf treatment	4160 ± 286 *	11.9 ± 0.8 *	88 ± 16 *	25 ± 8 *	42 ± 3 *	13 ± 6 *

Legend: red blood cells (RBC), hemoglobin (Hb), total serum iron (TSI), serum ferritin (sFtn).

**Table 3:** Hematological values of 156 inflamed pregnant women affected by hereditary thrombophilia treated for 30 days with 100 mg of bovine lactoferrin two times a day before meals. Statistical analysis was performed by ANOVA. * Significant differences (*p* < 0.0001).

Serum Parameters	Mean Values ± Standard Deviation					
	RBC 10^3^/mmc	Hb g/dL	TSI µg/dL	sFtn ng/mL	Hematocrit %	IL-6 pg/mL
Before bLf treatment	3860 ± 214	10.4 ± 0.8	60 ± 18	15 ± 7	28 ± 4	94 ± 7
After bLf treatment	4150 ± 75 *	12.5 ± 0.3 *	94 ± 7 *	32 ± 4 *	36 ± 9	48 ± 12 *

Legend: red blood cells (RBC), hemoglobin (Hb), total serum iron (TSI), serum ferritin (sFtn).

**Table 4:** Hematological values of 249 pregnant women suffering from IDA treated for 30 days with 520 mg of ferrous sulfate once a day during meals. Statistical analysis was performed by ANOVA. * Significant differences (*p* < 0.0001).

Serum Parameters	Mean Values ± Standard Deviation					
	RBC 10^3^/mmc	Hb g/dL	TSI µg/dL	sFtn ng/mL	Hematocrit %	IL-6 pg/mL
Before ferrous sulfate treatment	3705 ± 162	10 ± 0.7	37 ± 10	15 ± 5	29 ± 6	33 ± 9
After ferrous sulfate treatment	3745 ± 123	11 ± 1.0	47 ± 11	14 ± 4	29 ± 3	52 ± 13

Legend: red blood cells (RBC), hemoglobin (Hb), total serum iron (TSI), serum ferritin (sFtn).

**Table 5:** Clinical parameters and IL-6 levels in gingival crevicular fluids before and after treatment with orosoluble tablets, containing 50 mg of bovine lactoferrin, three times a day after accurate oral hygiene on 30 out of 50 enrolled volunteers. The clinical trial and statistical analysis are in progress.

Clinical Parameters	Mean Values ± Standard Deviation					
	PPD	GI	PlI	BOP (%)	CAL (mm)	IL-6 (ng/mL)
Baseline	2.8 ± 0.3	0.80 ± 0.10	0.80 ± 0.10	32	1.50 ± 0.60	1.42 ± 0.30
After 4 weeks of bLf treatment	0.6 ± 0.7	0.50 ± 0.10	0.40 ± 0.20	0	0.50 ± 0.30	0.55 ± 0.31

Legend: probing pocket depth (PPD), gingival index (GI) plaque index (PlI), bleeding on probing (BOP), clinical attachment level (CAL).
